# Functional MRI To Evaluate “Sense of Self” following Perforator Flap Breast Reconstruction

**DOI:** 10.1371/journal.pone.0049883

**Published:** 2012-11-27

**Authors:** Stephanie A. Caterson, Sharon E. Fox, Adam M. Tobias, Bernard T. Lee

**Affiliations:** 1 Brigham and Women’s Hospital, Harvard Medical School, Boston, Massachusetts, United States of America; 2 Department of Surgery, Division of Plastic and Reconstructive Surgery, Beth Israel Deaconess Medical Center, Harvard Medical School, Boston, Massachusetts, United States of America; 3 Division of Health Sciences and Technology, Massachusetts Institute of Technology, Cambridge, Massachusetts, United States of America; Northwestern University Feinberg School of Medicine, United States of America

## Abstract

**Background:**

Breast reconstruction is associated with high levels of patient satisfaction. Previous patient satisfaction studies have been subjective. This study utilizes functional magnetic resonance imaging (fMRI) to objectively evaluate “sense of self” following deep inferior epigastric perforator (DIEP) flap breast reconstruction in an attempt to better understand patient perception.

**Methods:**

Prospective fMRI analysis was performed on four patients before and after delayed unilateral DIEP flap breast reconstruction, and on four patients after immediate unilateral DIEP flap breast reconstruction. Patients were randomly cued to palpate their natural breast, mastectomy site or breast reconstruction, and external silicone models. Three regions of interest (ROIs) associated with self-recognition were examined using a general linear model, and compared using a fixed effects and random effects ANOVA, respectively.

**Results:**

In the delayed reconstruction group, activation of the ROIs was significantly lower at the mastectomy site compared to the natural breast (p<0.01). Ten months following reconstruction, activation of the ROIs in the reconstructed breast was not significantly different from that observed with natural breast palpation. In the immediate reconstruction group, palpation of the reconstructed breast was also similar to the natural breast. This activity was greater than that observed during palpation of external artificial models (p<0.01).

**Conclusions:**

Similar activation patterns were observed during palpation of the reconstructed and natural breasts as compared to the non-reconstructed mastectomy site and artificial models. The cognitive process represented by this pattern may be a mechanism by which breast reconstruction improves self-perception, and thus patient satisfaction following mastectomy.

## Introduction

The psychological benefits of breast reconstruction have long been touted in the plastic surgery literature, including high patient satisfaction and quality of life, improved perception of physical appearance, as well as return of a feeling of normalcy and wholeness [1]–[5]. In addition, reconstruction of the breast after mastectomy has been shown to improve psychosocial morbidity and reduce post-operative depression [6]. Patient satisfaction has been demonstrated to be higher following autologous reconstruction, compared to implant based reconstruction [5], [7]. To date, reports of post-reconstruction patient satisfaction have been subjective in nature. Subjective studies have innate limitations, with uncontrolled variables in discernment from patient to patient.

Functional magnetic resonance imaging (fMRI) is a non-invasive technique for measuring brain activity by detecting changes in blood oxygenation and flow that occur in response to neural activity. More active areas consume more oxygen, increasing demand blood flow. Activation maps are then created showing which parts of the brain are associated with a particular action or task. Recognition of the body as “self” is a fundamental aspect of self consciousness.

Self-attribution of body parts is thought to be mediated by multisensory perceptual correlations [8]–[12]. Recent neuroimaging studies of the “rubber hand illusion” correlate a specific network of activity in the brain with perceived sensation in a fake hand [11]. These findings are particularly relevant when considering the optimal reconstruction of a body part lost to trauma or disease, as seen in breast cancer treatment.

The purpose of this study is to objectively evaluate self-recognition of deep inferior epigastric perforator (DIEP) flap breast reconstruction. We used fMRI to measure the activity of brain regions of interest (ROIs) previously associated with self-recognition (or “sense of self”), both before and after DIEP flap breast reconstruction to assess the multisensory perceptual correlates of breast reconstruction. Our hypothesis was that stimulation of the reconstructed breast would elicit brain activity patterns in the premotor cortex correlated with self-recognition and perceived sensation [4] and that these activity patterns would be similar to the natural breast.

## Patients and Methods

**Figure 1:**
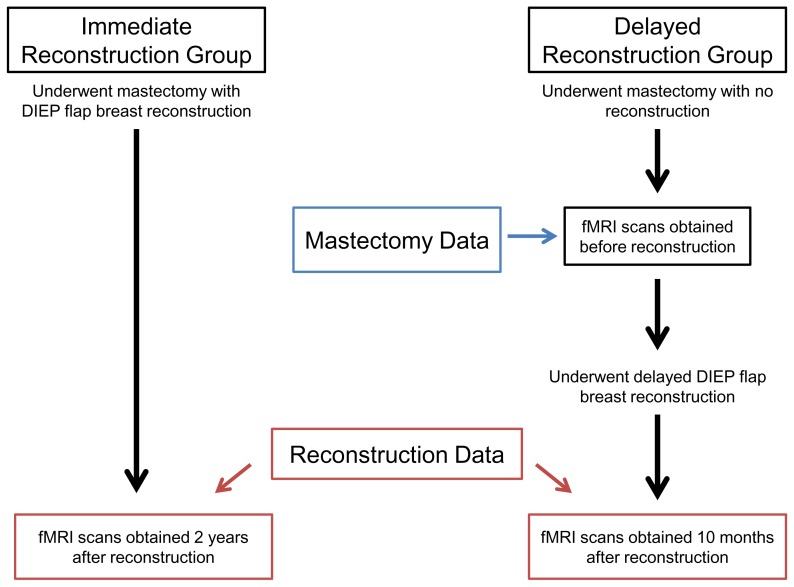
Timeframe for Data Collection. “Delayed-reconstruction” patients were imaged twice; once after mastectomy and no reconstruction and again after a delayed autologous breast reconstruction. “Immediate-reconstruction” patients were imaged once after a combined procedure consisting of unilateral mastectomy and immediate autologous breast reconstruction.

This study was approved by the Committee on Clinical Investigation at Beth Israel Deaconess Medical Center, and written informed consent was obtained from all patients. The experiments were conducted according to the Declaration of Helsinki. All patients presenting to our institution from 2004–2006 for autologous breast reconstruction were evaluated and patients meeting criteria were contacted. Participants were divided into two groups. The first group underwent mastectomy prior to DIEP flap reconstruction (n = 4). We imaged this “delayed-reconstruction” group after mastectomy and no reconstruction and again ten months after a delayed breast reconstruction. The second group had a simultaneous mastectomy and DIEP flap reconstruction (n = 4). We imaged this “immediate-reconstruction” group two years after reconstruction (Figure 1). All patients had a unilateral breast reconstruction and one unaffected breast. This unaffected (“natural”) breast served as an internal control for self-recognition.

### Patient Demographics

The average age for the delayed-reconstruction group was 43 years. The immediate-reconstruction group averaged 57 years. All patients were right-hand dominant and had one unaffected natural breast, allowing for comparison. The groups were balanced between left and right-sided operative sites (mastectomy in the case of the “delayed-reconstruction” group and DIEP flap breast reconstruction in the case of the “immediate-reconstruction group) so that effects of sidedness would be eliminated. The average time lapse from mastectomy to fMRI study was 28 months. The average time lapse from DIEP flap reconstruction in the “immediate-reconstruction” group to fMRI study was 37 months. The average time from DIEP flap reconstruction in the “delayed reconstruction” group to the follow-up fMRI was 10 months.

### Data Acquisition

Functional scans were acquired while patients were randomly cued to complete four tasks: 1) palpation of the natural, non-cancerous breast, 2) palpation of the mastectomy site or DIEP flap, 3) palpation of an “artificial” silicone gel implant model on the left side of the body, and 4) palpation of a silicone gel implant model on the right side of the body. These artificial implant models were placed next to the patient and used as a control for hand sensation, as well as a “non-self” object similar in shape to the natural breast. Prior to scanning, patients practiced moving their arms at the elbow in front of a mirror to achieve the task without head movement, and with the resting state of the arm approximately equidistant from the patient’s chest and hips. Foam padding was used to prevent head movement within the coil, and paper tape was placed across the forehead to provide further reference of head movement to the patient. Functional MRI data was acquired using the 3T Philips scanner. Patient cues were generated in random sequence using images displayed with Psyscope 1.2.5 [13]. Functional data analysis was performed using Brainvoyager QX software package (Brain Innovation, Maastricht, Netherlands).

**Figure 2:**
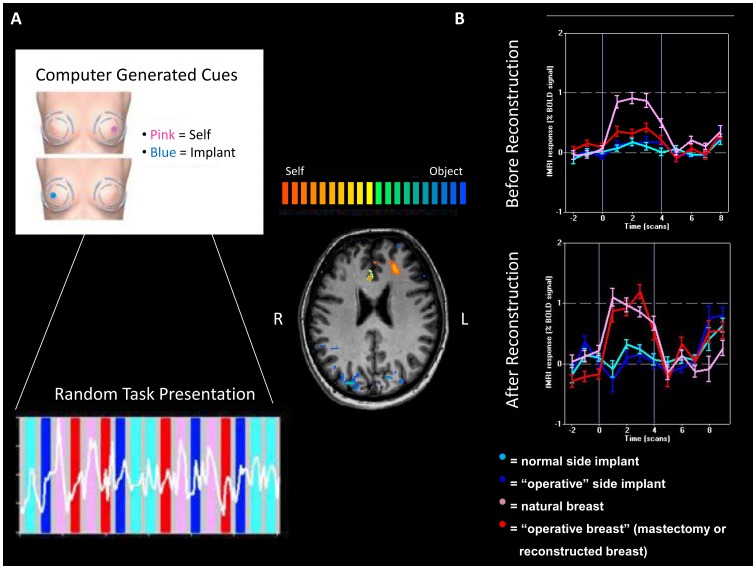
fMRI Response in Regions of Interest (ROI). A) Cues to palpate self (pink), or artificial model (blue) specified to side. Randomized block design alternates between palpation and rest. B) Cortical ROI of “self” (natural breast; pink) versus “non-self” (artificial model; blue/cyan). Increased activity observed following reconstruction (red).

Tasks were arranged using a randomized block design paradigm. Large limb movements could be observed via an infrared camera. Tasks were cued visually using a projected image of a circular pattern for breast self-exam (Figure 2A). A dot cue appeared on the breast image with one of two colors (blue/pink), and one of two locations (right or left breast), allowing for a 2×2 matrix model of the tasks for all patients (Figure 2A). Blue dots were used to cue palpation of the artificial models, and pink dots were used to cue palpation of the patient’s natural breast, DIEP flap reconstruction, or mastectomy site.

The side to be palpated was cued by right or left location of the dot. The color and location of the dot (right or left breast, from the patient’s perspective) occurred at random, and patients were instructed to mirror the side on which the dot appeared by touching their own breast or the breast model on that side of the body. Patients were instructed to use the hand ipsilateral to the breast indicated by the cue for palpation (i.e. –palpation of the left breast with the left hand, and palpation of the right breast with the right hand). By this method, we hoped to control for effects of the hand being used for stimulation. Rest occurred between each task, indicated by an “X” at the center of the screen, during which time the patient was instructed not to move except to return her hands to the baseline position. Images were displayed with Psyscope 1.2.5 [13]. The time for all tasks was 12 seconds. Baseline rest occurred for approximately 9 seconds between each task, with slight variation (<1 second) due to the time necessary for the patient to return her hands to the baseline position.

Artificial models were composed of a silicone gel implant (Mentor MemoryGel™) with cloth covering and a foam nipple constructed to represent the tactile surface of a breast. These were fixed to a foam board placed under the hips, so that each model could be easily palpated adjacent to the body. The models were employed to control for effects of hand sensation, and as a “non-self” object comparable to the breast.

### MRI Acquisition

The 3T Philips scanner used was equipped with 22 mT/m field gradients with a slew rate of 120 T/m/s (Echospeed). The pulse sequence used was the gradient-echo echo planar imaging (EPI) sequence. Three-dimensional anatomical volumes were collected using a high resolution T1 SPGR sequence. The functional MRI protocols were based on a multi-slice gradient echo, echo-planar imaging (EPI), using a standard head coil. Functional data was obtained under optimal timing parameters: TR = 3 sec, TE = 55 ms, flip angle = 90°, imaging matrix = 80×80, FOV = 24 cm. The 37 slices (slice thickness 4 mm and 0 mm gap) were oriented approximately to the axial plane and covering the whole brain.

### Data Analysis

Functional data analysis was performed using Brainvoyager QX software package (Brain Innovation, Maastricht, Netherlands). Talairach coordinates were applied to high resolution SPGR scans, allowing for normalization and comparison across all patients.

Preprocessing included head motion correction, slice scan time correction and high-pass temporal smoothing in the frequency domain to remove drifts and to improve signal to noise ratio. To compute statistical parametric maps we applied a general linear model.

(GLM) using predictors convoluted with a typical hemodynamic response function. Data from each patient group was combined, and analyzed using a multi-subject GLM. We analyzed the BOLD signal change of pre-determined ROIs (defined by Talairach coordinates) within subjects using the general linear model with four separate contrasts of the tasks, and a fixed effects analysis of variance (ANOVA). A fixed effects analysis was felt to be appropriate for this purpose as no hypothesis was being tested regarding the existence of these pre-determined regions. Instead, maximal sensitivity was desired for identifying their average location within each group of subjects, and for applying a more rigorous test within each subject. For comparisons across groups, a random effects ANOVA was employed. Significance levels were calculated taking into account the probability of a false detection for any given cluster (10×10×10 voxels). Minimum significance level was set at p<0.01, corrected for multiple comparisons.

### Time Course Analysis

The first contrast compared perception of the natural breast (+1) to the artificial model (−1) on the same side of the body. A second contrast compared perception of the mastectomy site or DIEP flap (+1) to the artificial model (−1). A final contrast compared perception of the natural breast (+1) to the DIEP flap or mastectomy site (−1). Activation patterns for each condition were averaged across trials, and subjects within each group.

The average percent signal change and standard errors were then calculated for each condition. The magnitude of activation was sampled from three ROIs. These regions were selected based upon predicted location of activity from previous studies identifying self-referential regions, and were the only regions with significantly different activation on random effects analysis.

## Results

**Figure 3:**
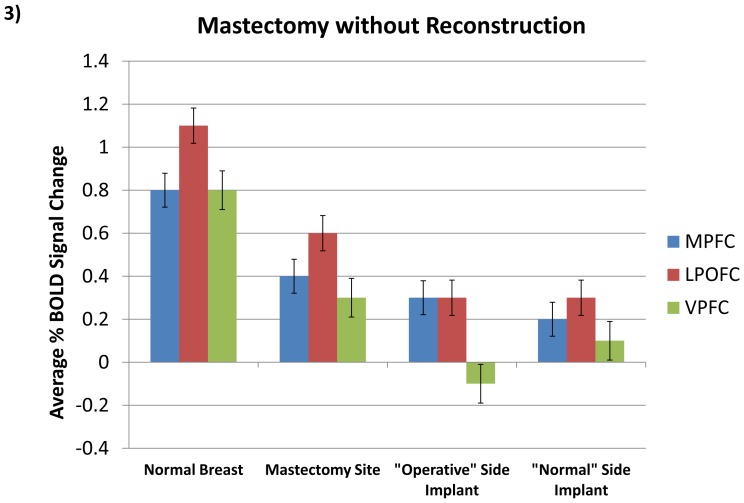
Comparative Results in Regions of Interest. Activity corresponding to normal breast, mastectomy site and artificial implant model (p<0.01, Bonf.). Bars indicate standard error.

The first analysis was between palpation of the natural breast and the mastectomy site with no reconstruction. In all patients, these three regions of significantly higher activity were identified with palpation of the natural breast in contrast to light touch of the mastectomy site. The ROIs were designated as the medial prefrontal cortex (MPFC, Tal: ×  = 4, y = 26, z = 25; t = 14.73, p<0.01, Bonf.; Figure 2B, 3), left posterior orbitofrontal cortex (LPOFC, × = −30, y = 35, z = 12, t = 5.09, p<0.01, Bonf.; Figure 3), and ventral prefrontal cortex (VPFC, Tal: × = −48, y = 12, z = 20; t = 10.47, p<0.01, Bonf.; Figure 3).

**Figure 4:**
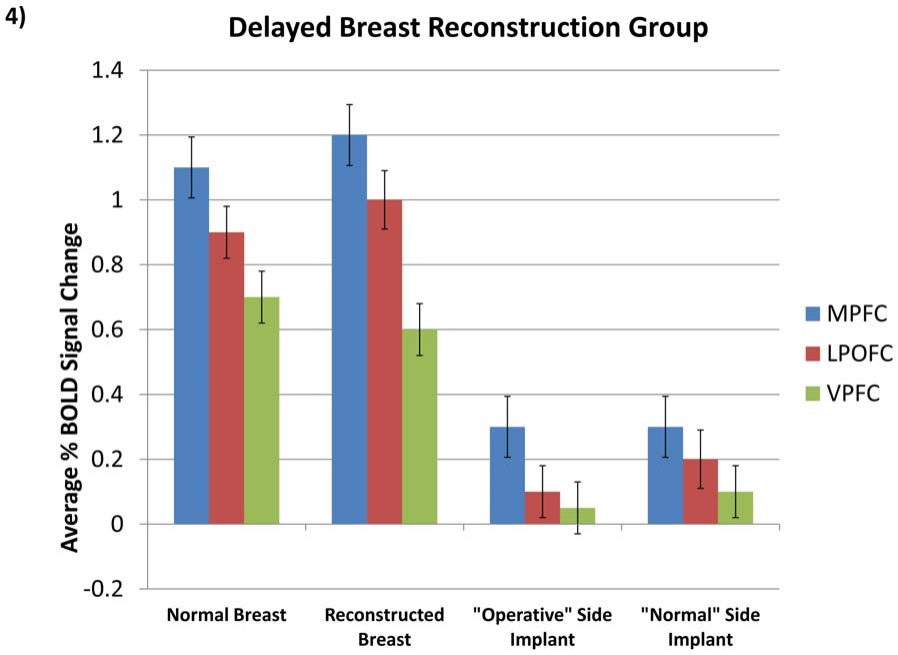
Comparative Results in Regions of Interest. Activity corresponding to normal and (delayed) reconstructed breast compared to models (p<0.01, Bonf.). Bars indicate standard error.

We then analyzed the same regions in these patients after delayed DIEP flap reconstruction ten months later. These patients exhibited significantly greater activity in the MPFC (t = 6.63, p<0.01, Bonf.; Figure 4), LPOFC (t = 8.22, p<0.01, Bonf.; Figure 4), and VPFC (t = 5.02, p<0.01, Bonf.; Figure 4) with light touch of the reconstructed breast in comparison to their previous mastectomy site. This difference in response to the mastectomy site was noted in all three regions.

**Figure 5:**
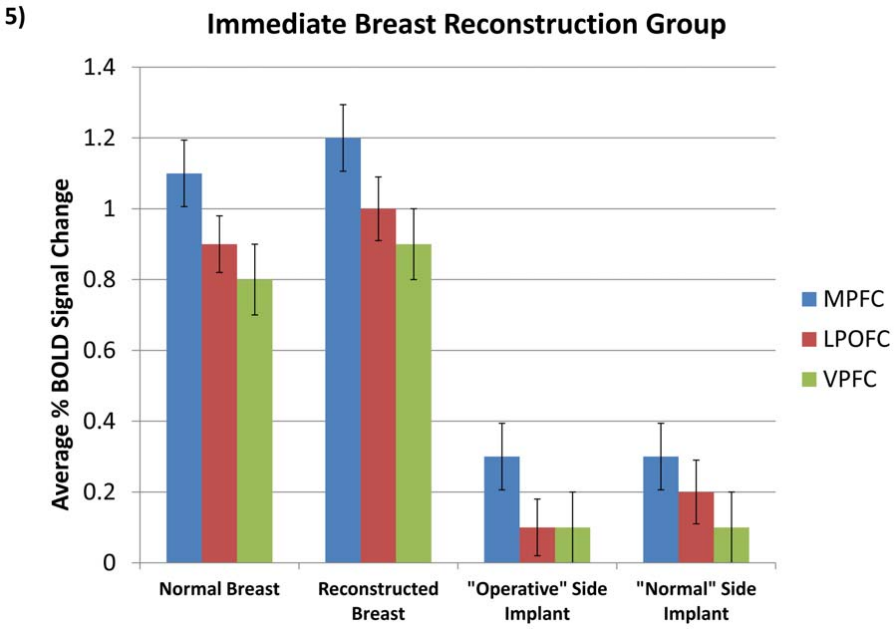
Comparative Results in Regions of Interest. Activity corresponding to normal and (immediate) reconstruction compared to models (p<0.01, Bonf.). Bars indicate standard error.

Next, we compared the DIEP flap reconstructed breast to non-self (the corresponding artificial implant model). In the immediate-reconstruction group, reconstructed breasts had significantly higher activity in comparison to the artificial models. This was seen in the MPFC (t = 6.54, p<0.01, Bonf.; Figure 5), LPOFC (t = 7.61, p<0.01, Bonf.; Figure 5), and VPFC (t = 6.45, p<0.01, Bonf.; Figure 5). No significant difference was observed in these regions between the delayed-reconstruction and immediate-reconstruction groups.

Finally, we compared perception of self (the natural breast) to non-self (the artificial implant model on the same side of the body). In all patients, palpation of the natural breast revealed significantly higher activity in three regions in contrast to palpation of artificial models. This activity was seen in the MPFC (t = 4.82, p<0.01, Bonf.), LPOFC (t = 7.77, p<0.01, Bonf.) and VPFC (t = 7.61, p<0.01, Bonf.). This pattern was consistent within each patient group, as well as averaged across both groups.

In the delayed-reconstruction group, palpation of the mastectomy site was associated with activation of the anterior cingulate, though this was not observed in the immediate-reconstruction group. Also noted in the delayed-reconstruction group was activation of primary sensory cortex in response to palpation of the artificial implant model. This is likely due to increased tactile stimulation of the hand by the artificial breast and nipple as compared to the skin overlying the mastectomy site.

## Discussion

The main focus of this study was to examine self-attribution, or the sense of the reconstructed DIEP flap breast as a true body part rather than a prosthesis. We found significant activation of the MPFC, LPOFC, and VPFC regions associated with palpation of the natural breast and the reconstructed breast in contrast to the artificial models or the mastectomy site. The prefrontal cortex has been previously described in association with an active state of being, or preparation for motor activity, as well as assumption of the “first person perspective” [14]. The MPFC has been noted across the literature to be differentially active in association with self-perception. The same has been shown to be true of the LPOFC [14]. MPFC activity has been noted to be a critical component of self- reflection, and in particular self-referential thoughts or memories [15].

Interestingly, in the delayed-reconstruction patient group, MPFC activity was greater in response to palpation of the natural breast than in response to the mastectomy site. This difference becomes more pronounced when examining the immediate reconstruction group, where palpation of the reconstructed breast and the natural breast resulted in similar activation of the MPFC. Such differences may be due to a perceptual effect of mirroring the projected task, where patients with a reconstructed breast perceived greater symmetry with the visual pictured exam task (expected self) than patients without reconstruction. The MPFC was not active in response to palpation of the artificial models, however, which leads to the possibility that self-reference, or recognition of the reconstructed breast as a body part is involved.

The VPFC has also been implicated in the multisensory representation of one's own body [11]. As suggested by Ehrsson et al. in the “rubber-hand” phenomenon [11]–[12], premotor neurons represent both the seen and felt position of the body and discharge when a “self” attributed part is touched and when a visual stimulus is presented near the body [16]–[19]. The fact that “false” self-attribution in the rubber hand phenomenon occurs specifically when tactile or visual input is synchronous with “self” sensory input suggests that multisensory synchrony may be the key to achieving self-attribution of an artificially reconstructed body part.

Activity in response to the rubber hand illusion has been noted in the bilateral dorsal premotor cortex – consistent with the response to palpation of the reconstructed breast [11]. Several of these areas are known to be involved in the processing of proprioceptive signals, and may be involved in the “recalibration” of body position following initiation of the rubber hand illusion [9], [20]. It is possible that the same recalibration takes place following successful reconstruction of the body. Unlike these reports, parietal lobe activation was not significant in palpation of the natural or reconstructed breast. This difference may be due to the decreased role of peripheral sensory information from the insensate reconstructed breast in the attribution of “self,” as the location of the reconstructed breast is the same as that of the original breast prior to mastectomy. Our results mirror those of Ehrsson et al. in the association of activity in the premotor cortex with the feeling of ownership of a non-sensate, reconstructed breast [11]–[12]. We suggest that the congruence of expected tactile information (determined from the natural breast) with that received from the reconstructed breast is the underlying mechanism of self-attribution.

Previous studies evaluating breast reconstruction outcomes are subjective, focusing on patient satisfaction obtained through surveys. These studies have shown a uniformly high satisfaction rate with autologous reconstruction in comparison to implant reconstruction [5], [7]. Although patient satisfaction is quickly becoming an important measure of health care quality, there are limitations to these studies, including survey design and responder bias. Here we provide objective evidence that patients who undergo DIEP flap reconstruction exhibit a self-attribution of the reconstructed breast that is similar to a natural breast. We propose that this may be a means by which these procedures improve patient satisfaction and quality of life. By contrast, patients who undergo a mastectomy without reconstruction do not exhibit the same neural activation patterns, and this may underlie elements of patient dissatisfaction and psychosocial morbidity. Furthermore, when these same patients eventually proceed with delayed reconstruction, there is a reversal and restoration of the normal perceptual response. Our findings suggest that breast reconstruction can restore patterns of neural activity consistent with a feeling of ownership and “sense of self” in patients who require a mastectomy.

### Conclusion

This study is unique in that it is an objective evaluation of patient’s perception of perforator flap breast reconstruction results. “Sense of self” ROIs demonstrated by fMRI are active and similar to the natural breast after DIEP flap reconstruction two years post operatively. Patients who chose DIEP flap breast reconstruction can objectively demonstrate self-recognition of the reconstructed breast. “Sense of self” may be a mechanism by which breast reconstruction improves patient satisfaction and quality of life.
